# Knowledge about Computer Vision Syndrome among Bank Workers in Gondar City, Northwest Ethiopia

**DOI:** 10.1155/2020/2561703

**Published:** 2020-04-14

**Authors:** Getasew Alemu Mersha, Mohammed Seid Hussen, Gizachew Tilahun Belete, Melkamu Temeselew Tegene

**Affiliations:** Department of Optometry, College of Medicine and Health Sciences, University of Gondar, Ethiopia

## Abstract

**Background:**

Globally, computer vision syndrome is the most common eye problem which is associated with prolonged exposure to a computer. It has a great socioeconomic impact on the users due to its effect on job performance. Recently, many people in the world, including our country Ethiopia, especially bank workers, are spending most of their time in front of a computer screen to facilitate their work. Since it is assumed that knowledge is pertinent to prevent computer vision syndrome, this study was aimed at assessing the understanding of bank workers towards computer vision syndrome.

**Methods:**

An institution-based cross-sectional study was conducted on 248 bank workers. The study participants were selected by using a simple random sampling technique. A self-administered questionnaire was used to collect the data from the bank workers. The data was entered and analyzed by SPSS version 21 software.

**Results:**

A total of 248 bank workers completed the questionnaire fully, which was a response rate of 93.5% (*n* = 234). Among them, 155 (66.20%) were males and the majority of the respondents were in the age group of 20-39 years (223, 95.3%). The study revealed that from 234 respondents, 211 (90.20%) were aware of computer vision syndrome, while 26.9% of them had a good knowledge about the disorder. Majority of the respondents (40, 17.1%) cited mass media as a source of information.

**Conclusion:**

Even though the majority of computer-using bank workers heard of computer vision syndrome, it was recognized that only a small portion of the participants had good knowledge.

## 1. Introduction

Computer vision syndrome describes a group of eye and vision-related problems due to prolonged usage of computers. Most common symptoms related to computer vision syndrome are eye strain, blurred vision, headache, dry eye, backache, and neck and shoulder pain [1].

Nearly 60 million people suffer from computer vision problems globally, and a million new cases occur each year [2]. According to the National Institute of Occupational Safety and Health, computer vision syndrome affects about 90% of the people who spend three or more hours daily at a computer [3].

According to a study conducted to assess the prevalence and associated factors of computer vision syndrome among bank workers in Gondar city, Northwest Ethiopia, the prevalence of computer vision syndrome was 73% [4]. Computer vision syndrome decreases work productivity by 4% up to 19% [5].

The extent to which individuals experience visual symptoms often depends on the level of their visual abilities and the amount of time spent looking at the computer [6]. The predisposing factors for computer vision syndrome are uncorrected refractive error, inadequate eye focusing, aging changes, the level of contrast, the presence of glare and reflection on the screen, improper positioning, and viewing distance and angles [7].

Prevention or reduction of computer vision syndrome involves using appropriate glasses and/or contact lenses designed to meet unique visual demands of computer viewing and applying proper ergonomic modifications such as proper body positioning while using a computer, keeping the reference materials as close as possible to the screen, eliciting the room light and changing contrast and brightness of the screen, taking a brake every 20 minutes for 20 seconds, applying frequent blinking, and using lubricating eye drops [7].

Many studies have been done regarding the prevalence and associated factors of computer vision syndrome in the world and in our country Ethiopia. However, little is known about the awareness and knowledge of the disorder, so our study was conducted to determine the awareness and knowledge towards computer vision syndrome among bank workers in Gondar city, Northwest Ethiopia.

## 2. Methods

### 2.1. Study Design and Settings

An institutional-based cross-sectional study was conducted in Gondar city among bank workers, from April 15, 2019, to May 1, 2019. The area of the study was Gondar city, Northwest Ethiopia. Data obtained from the Gondar town administration statistical office indicated that Gondar city is the capital of the North Gondar Administration zone, in the Amhara region, situated 748 km from the capital city, Addis Ababa. According to the Gondar financial administration office, there are 26 governmental (commercial banks) and 10 private banks in the city. In these banks, there are around 540 bank workers who are working on a computer.

### 2.2. Sample Size Determination and Sampling Technique

Sample size was determined using a single population proportion formula by taking 50% proportion of good knowledge, 95% confidence level, 5% margin of errors, and 10% nonresponse rate. Accordingly, the final computed sample size was 248. A systematic random sampling technique was employed. To ensure representativeness, the sample was taken from the whole 36 banks. The calculated sample size was proportionally allocated to each bank, and then the study participants were selected using a simple random sampling technique.

### 2.3. Data Collection Procedure and Quality Control

The data was collected using a structured self-administered questionnaire which was modified and adopted from reviewed literatures [2, 8–10]. The questionnaire consisted of 16 items (5 sociodemographic, 1 spectacles use, 1 source of information, and 9 knowledge questions) and is attached as a supplementary material. The questionnaire was validated by doing a pretest on 5% of the sample before the actual data collection period. Necessary modification of the questionnaires was carried out based on the pretest feedback. The reliability of the questionnaires was checked, and their Cronbach alpha value was 0.83, and then the structured questionnaire was directly administered to the bank workers that were selected according to the simple random sampling technique.

Finally, the distributed questionnaires were collected from the bank workers after they completed the questionnaire. The participant's knowledge was assessed using 9 questions, and they were composed to estimate the overall knowledge. Finally, the overall knowledge was categorized using modified Bloom's cut-off point as good if the score was between 80 and 100%, moderate if the score was between 60 and 79%, and poor if the score was less than 60%. Modified Bloom's cut-off point is used to grade performance of knowledge based on how many percent of a test or a task is answered or done correctly. Based on this assumption, someone has a good knowledge if he/she does 80-100% of the task or questions, moderate knowledge if he/she does 60-79%, and poor knowledge if he/she does <60% [11].

### 2.4. Data Processing and Analysis

The collected data was entered and analyzed by SPSS version 21. Mean, median, and standard deviation were used to describe the data. A chi-squared test was applied to test the association between categorical variables at the level of significance (*P* value of <0.05). Moreover, the findings were presented by tables and a bar graph.

### 2.5. Ethical Clearance

Ethical clearance was obtained from the ethical review board of the University of Gondar College of Medicine and Health Science School of Medicine Ethical Review Committee. Hence, as there was a possibility of communicating with the participants, no waiver grant was needed. After the purpose of the study was explained to the participants, each of them was politely requested to participate, and verbal informed consent was obtained. Moreover, the study subjects were granted full right to participate or withdraw whenever they wanted to. Confidentiality of the collected information was assured through omission of any identifiers and storage in a secured folder.

## 3. Results

A total of 234 bank workers completed the questionnaire fully which represented a response rate of 94.35%. Out of 234 participants, 155 (66.2%) were male. The median age of respondents was 27. The majority of the respondents (146, 62.4%) had worked for less than five years, and 35 (15.0%) respondents had correction spectacles (Table 1).

Among 211 respondents who heard about computer vision syndrome, 63 (26.9%) of the respondents had good knowledge, 76 (32.5%) of the respondents had fair knowledge, and the majority of the respondents (95, 40.6%) had poor knowledge about computer vision syndrome (Table 2).

On applying the chi-squared test, working experience and knowledge about computer vision syndrome showed a significant association (*χ*2 = 10.926, df = 2, *P* value = 0.004). However, age, gender, educational status, and spectacles user were not significantly associated with knowledge about computer vision syndrome (*P* value > 0.05) (Table 3).

**Figure 1:**
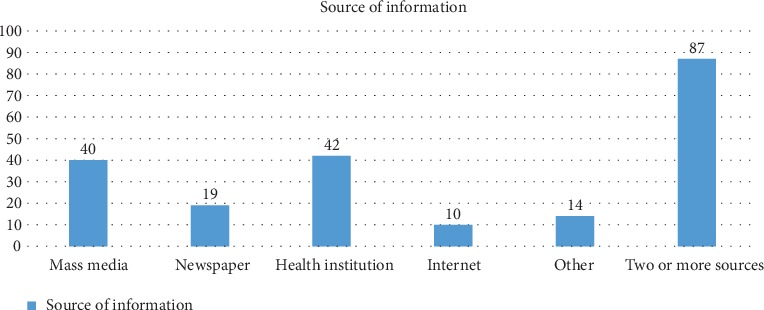
A bar graph showing the sources of information for the participants who have heard about computer vision syndrome (*n* = 211).

The majority of the respondents heard about computer vision syndrome from two or more sources, while most of the other respondents used mass media and health institution as sources of information for their awareness about computer vision syndrome. On the other hand, a few of the respondents used the internet and any other source (Figure 1).

## 4. Discussion

In this study, 90.2% had awareness about computer vision syndrome, which is comparable with the study done in Nairobi, Kenya (89.95) [12] and lower than the studies done in Abuja, Nigeria, among computer users (40%) [13] and ophthalmologists (66.7%) [14], and in Malerkotla, District Sangrur, and Punjab, India (74%) [2] and Karnataka, India (38.8%) [10]. However, it was lower than the study done in India among ophthalmologists who were computer users who claimed that all of the 134 respondents were aware of computer vision syndrome [15]. The difference is likely due to the difference in the study population characteristics.

Regarding the detailed knowledge of computer vision syndrome, only 32.5% had fair knowledge and 26.9% had good knowledge of computer vision syndrome which was higher than the study done in Ahmadabad, India, in which only 8.33% of the students had good knowledge [16] and in Bandung, Indonesia, in which only 25.6% of the respondents had fair knowledge of computer vision syndrome [17]; moreover, this result was consistent with the study done in Nigeria in which 27% of computer users had good knowledge on computer vision syndrome [13], and in Maharashtra, India, the majority of computer operators had poor knowledge on computer vision syndrome [18]. However, it was lower than the study done in Malaysia (51.20%) [8]; in Punjab, India, in which 74% of bank employees had knowledge of computer vision syndrome [2]; and in India in which almost all of the ophthalmologists knew about computer vision syndrome [15].

Overall, this comparison implicates that comprehensive information through education and training is pertinent to alleviate the vision problem secondary to computer vision syndrome.

Regarding the preventive measure for computer vision syndrome in this study, 29.5% had good knowledge on the preventive mechanism of computer vision syndrome which was lower than the study done in Malaysia where 64.4% of the administrative staffs had good knowledge on preventive measure on computer vision syndrome [19].

In this study, it was found that there was no significant association between age, gender, educational status, and spectacles users with knowledge on computer vision syndrome. However, there was a significant association between working experience and the knowledge on computer vision syndrome (*P* value < 0.05). The possible reason is that the respondents who had lots of experience would have prioritized knowledge on computer vision syndrome through different circumstances like taking part in conference and trainings on occupational hazards of electronics. Extensive literature search did not reveal many publications on the knowledge of computer vision syndrome, thus making comparison with other results difficult, and analytical components were not incorporated in this study, so that we did not identify the associated factors for the dependent variables.

## 5. Conclusion

Even though the majority of computer-using bank workers were aware of computer vision syndrome, the overall knowledge on the disorder was poor. It is recommended that further studies be carried out on a large scale to determine the extent of the computer vision syndrome problem among employees at workplaces including schools, colleges, higher education institutions, government departments, and the private sector in Ethiopia.

## Figures and Tables

**Table 1:** Sociodemographic characteristics of the bank workers in Gondar city, Northwest Ethiopia, 2019 (*N* = 234).

Variables		Frequency	Percent (%)
Gender	Male	155	66.2
	Female	79	33.8

Age	20-39	223	95.3
	≥40	11	4.7

Educational status	Diploma	30	12.8
	First degree	181	77.4
	Second degree	23	9.8

Working experience	<5 years	146	62.4
	≥5 years	88	37.6

Spectacles use	Yes	35	15.0
	No	199	85.0

**Table 2:** Participant's knowledge about computer vision syndrome in Gondar city, Northwest Ethiopia (*N* = 211).

No.		Response		
	Questions	Poor	Fair	Good
1	What are the causes of CVS?	55 (23.5%)	78 (33.3%)	101 (43.2%)
2	What are the symptoms of CVS?	159 (67.9%)	57 (24.4%)	18 (7.7%)
3	What are the prevention mechanisms of CVS?	71 (30.3%)	94 (40.2%)	69 (29.5%)
	Overall knowledge (*n* = 234)	95 (40.6%)	76 (32.5%)	63 (26.9%)

**Table 3:** Chi-squared test showing association between knowledge about computer vision syndrome and socioeconomic factors among bankers, Gondar city, Northwest Ethiopia, 2019 (*N* = 211).

Variables	Knowledge of CVS			*χ*2	df	*P* value
	Good		Poor			
Age						
20-39	55	129	39	2.621	2	0.270
≥40	5	4	2			
Gender						
Female	44	83	28	2.301	2	0.316
Male	16	50	13			
Educational status						
Diploma	7	19	4	7.160	4	0.128
First degree	42	105	34			
Second degree	11	9	3			
Working experience						
<5 years	27	89	30	10.92	2	0.004^∗^
≥5 years	33	41	11			
Spectacles user						
Yes	12	14	9	4.829	2	0.089
No	48	119	32			

^∗^Significant *P* value < 0.05.

## Data Availability

The data collection tools and the SPSS data used to support the findings of this study are currently under embargo while the research findings are commercialized. Requests for data, 6 months after publication of this article, will be considered by the corresponding author.
