# Supplementary material for: Knowledge about Computer Vision Syndrome among Bank Workers in Gondar City, Northwest Ethiopia
Source: Occup Ther Int. 2020 Apr 14;2020:2561703. doi: 10.1155/2020/2561703 (PMC7195649; doi:10.1155/2020/2561703)
Supplement: Supplementary Materials [file 2561703.f1.docx]

**Questionnaire on Knowledge about computer vision syndrome**

Questionnaire for assessment of knowledge of computer vision syndrome among bank worker computer users in Gondar town. The correct answers for the knowledge questions are highlighted.

**Part I: socio-demographic data**

1. Age: ____________ (years)

2. Sex: A) Male B) Female

3. Religion: A) Orthodox

B) Muslim

C) catholic

D) Protestant

E) Others______

4. Educational status: A) Certificate C) Degree

B) Diploma D) Masters and above

5. Working experience: __________ (years)

6. Spectacle or lens use: A) Yes B) No

**Part 2: Knowledge about Computer Vision Syndrome**

7. Have you ever heard of computer vision syndrome? A) Yes B) No

8. If you say **“yes”** for question 7, do you know about computer vision syndrome?

A) Yes B) No

9. If your answer is “**Yes”** for question 8, what do you understand by computer?

vision syndrome?

1. Tiredness during computer use
2. Use computer excessively

C) Neck pain during computer use

D) Combination of headache, eyestrain and blurring of vision that occur as

result of prolonged computer use

10. How did you get to know about Computer Vision Syndrome?

(Encircle whichever applicable)

A) Friend/Relative/Colleague

B) Mass media

C) Internet

D) Health institutions

E) Others (specify) __________________

11. Do you know about the symptoms of computer vision syndrome? A) Yes B) No

12. If your answer is “**yes”** for question 11, what do you think are the symptoms of

computer vision syndrome :( Tick more than one whenever applicable)

A) Pain [ ]

B) Irritation [ ]

C) Blurred vision [ ]

D) Headache [ ]

E) Neck pain [ ]

F) Eyestrains [ ]

G) Dryness of the eyes [ ]

H) Double vision [ ]

13. Do you know the cause of computer vision syndrome? A) Yes B) No

14. If your answer is “**yes**” for question 13, what is the cause of computer vision syndrome? (Tick more than one whenever applicable)

A) Prolonged glaring at the computer screen

B) Too bright computer screen

C) External light reflection on the computer screen

D) Poor eyesight

E) Working very close to the computer

15. Do you know about prevention measures of computer vision syndrome?

A) Yes B) No

16. If your answer is **“yes”** to question 15, what would you consider being

important prevention measure of computer vision syndrome?

(Tick more than one whenever applicable)

A) Taking regular breaks

B) Blinking frequently

C) Checking eyes regularly

D) Using glare screen on the computer.

**Thank you!**
